# Ischemic Hepatopathy Without Shock: A Complication of Pancreatitis

**DOI:** 10.7759/cureus.112343

**Published:** 2026-07-09

**Authors:** Orlando D Palmer, Elsie N Kodjoe, Mohamad Rezek, Oluwamisimi Abib, Walter Agyeman

**Affiliations:** 1 Internal Medicine, Piedmont Athens Regional Medical Center, Athens, USA

**Keywords:** acute liver injury, acute pancreatitis, extreme transaminitis, hemophagocytic lymphohistiocytosis mimic, hlh mimic, ischemic hepatopathy

## Abstract

While ischemic hepatopathy is most commonly associated with systemic hypotension or cardiac failure, it may also occur in the setting of microvascular compromise secondary to severe systemic inflammation. Ischemic hepatopathy is characterized by acute, marked elevations in aminotransferase levels with minimal cholestasis and rapid biochemical recovery. Although acute pancreatitis is a recognized trigger of disseminated intravascular coagulation (DIC), hepatic ischemia resulting from this complication is rarely reported. We present the case of a 49-year-old man with acute-on-chronic pancreatitis who developed extreme transaminase elevation (aspartate aminotransferase (AST) > 10,000 IU/L), severe thrombocytopenia, and coagulopathy in the absence of hypotension or hepatic encephalopathy. An extensive evaluation excluded viral, autoimmune, toxic, metabolic, and obstructive etiologies, as well as hemophagocytic lymphohistiocytosis (HLH). Liver enzyme levels improved rapidly with supportive care and N-acetylcysteine therapy. This case highlights pancreatitis-associated ischemic hepatopathy as an important diagnostic consideration in patients presenting with marked aminotransferase elevation.

## Introduction

Ischemic hepatopathy, also referred to as shock liver, ischemic hepatitis, or hypoxic hepatitis, is characterized by a sudden and marked elevation in serum aminotransferase levels, typically occurring within 48 hours, accompanied by relatively modest bilirubin elevation and rapid biochemical resolution within seven days once hepatic perfusion is restored and other causes of liver injury have been excluded. Although classically associated with cardiogenic shock, septic shock, or respiratory failure, ischemic hepatopathy may also occur in the absence of sustained hypotension, particularly in the setting of systemic inflammation and microvascular thrombosis [1,2].

Most studies define ischemic hepatopathy as a sudden increase in serum aminotransferase levels of at least 15-20 times the upper limit of normal in an appropriate clinical setting. Aspartate aminotransferase (AST) and alanine aminotransferase (ALT) levels exceeding 5,000-10,000 IU/L strongly favor ischemic hepatopathy over most other causes of acute liver injury. The mechanism of hepatic injury is primarily metabolic, resulting from oxygen deprivation rather than inflammation, although it may occur in the setting of a systemic inflammatory response [1,2].

Acute pancreatitis is a systemic inflammatory disorder that can precipitate multiorgan dysfunction and coagulation abnormalities, including disseminated intravascular coagulation (DIC) [3-5]. Although DIC is a recognized complication of severe pancreatitis, ischemic hepatopathy secondary to pancreatitis-associated coagulopathy has rarely been reported. DIC can result in microvascular thrombosis within the hepatic sinusoids, impairing hepatic perfusion and leading to ischemic hepatopathy, which is characterized by marked elevations in serum aminotransferase levels followed by progressive increases in bilirubin and the international normalized ratio (INR) [6-8]. Ischemic hepatopathy and pancreatitis-associated DIC are further linked through a bidirectional, self-amplifying pathophysiologic cycle in which microvascular thrombosis, hepatic synthetic dysfunction, and systemic inflammation perpetuate and exacerbate one another [9,10].

We report the case of a patient with acute-on-chronic pancreatitis who developed extreme aminotransferase elevation and consumptive cytopenias, highlighting the diagnostic challenges in differentiating ischemic hepatopathy from acute liver failure, acetaminophen toxicity, and hemophagocytic lymphohistiocytosis (HLH).

## Case presentation

A 49-year-old man with chronic pancreatitis, ongoing alcohol use without evidence of acute intoxication, and diabetes mellitus diagnosed four months earlier presented to the emergency department with abdominal pain. He was diagnosed with acute-on-chronic pancreatitis and discharged with opioid analgesics. He reported chronic acetaminophen use but denied any supratherapeutic ingestion.

The patient returned within 24 hours with worsening abdominal pain. Laboratory investigations revealed marked hepatocellular injury, with ALT approximately 2,000 IU/L and AST exceeding 10,000 IU/L, compared with normal aminotransferase levels during the previous visit. Alkaline phosphatase was minimally elevated, while pancreatic enzyme levels remained unchanged. Total bilirubin remained stable at approximately 2 mg/dL.

He subsequently developed abrupt and severe thrombocytopenia, with the platelet count declining from 361 × 10⁹/L to 8 × 10⁹/L, accompanied by prolonged prothrombin time, prolonged activated partial thromboplastin time, and worsening anemia. Physical examination revealed purpura and subconjunctival hemorrhage. He remained hemodynamically stable throughout the admission and did not develop hepatic encephalopathy.

The patient was admitted to the intensive care unit. Gastroenterology and hematology/oncology consultations were obtained. Liver transplant evaluation was initiated but was deferred because of active pancreatitis and severe thrombocytopenia. Given the concern for acute liver injury, intravenous N-acetylcysteine therapy was initiated.

**Figure 1:**
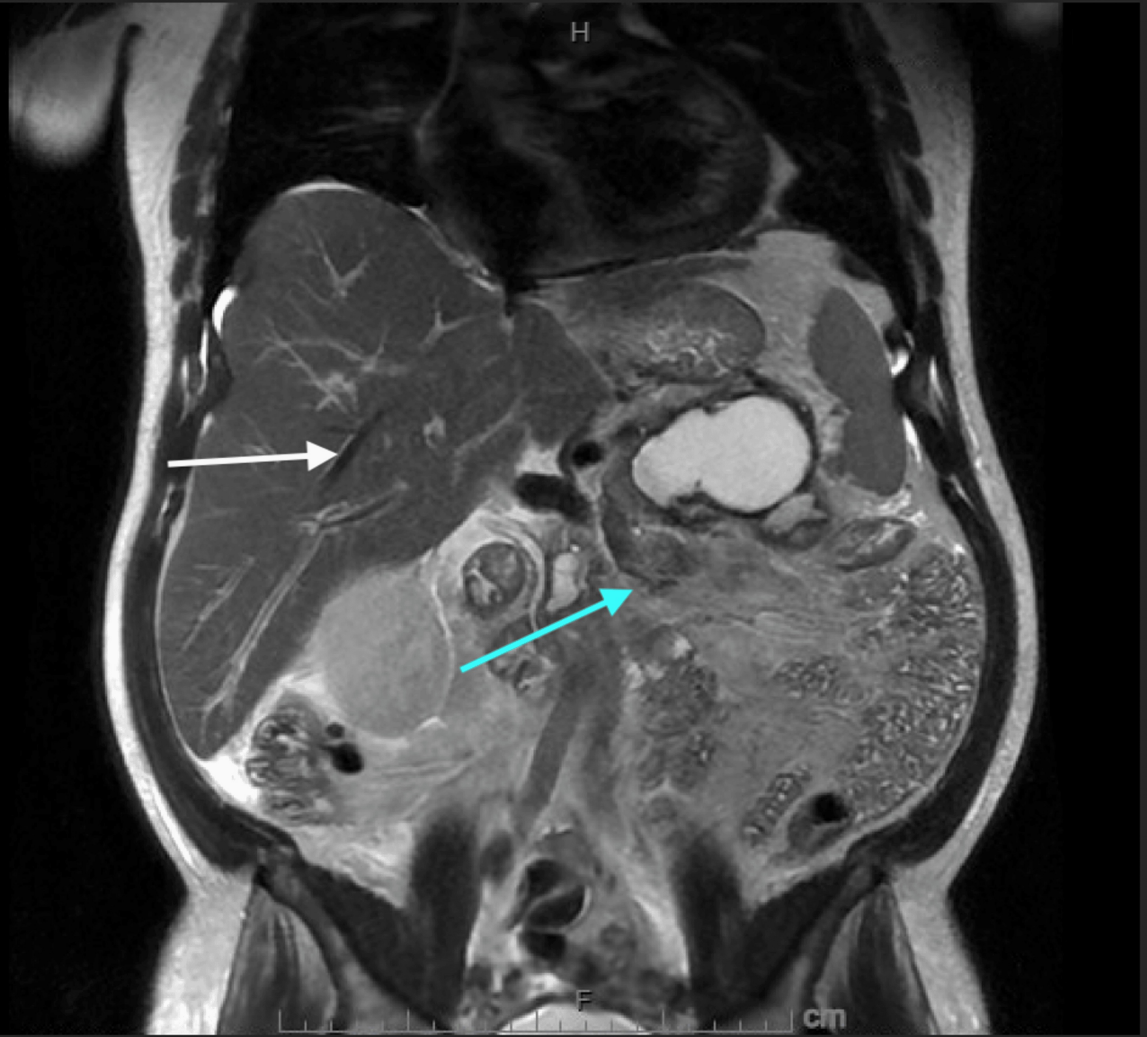
Coronal abdominal imaging demonstrating extensive pancreatitis with surrounding inflammatory fluid (blue arrow) and preserved hepatic vascular architecture (white arrow), supporting systemic inflammatory microvascular injury as the etiology of hepatocellular necrosis.

**Figure 2:**
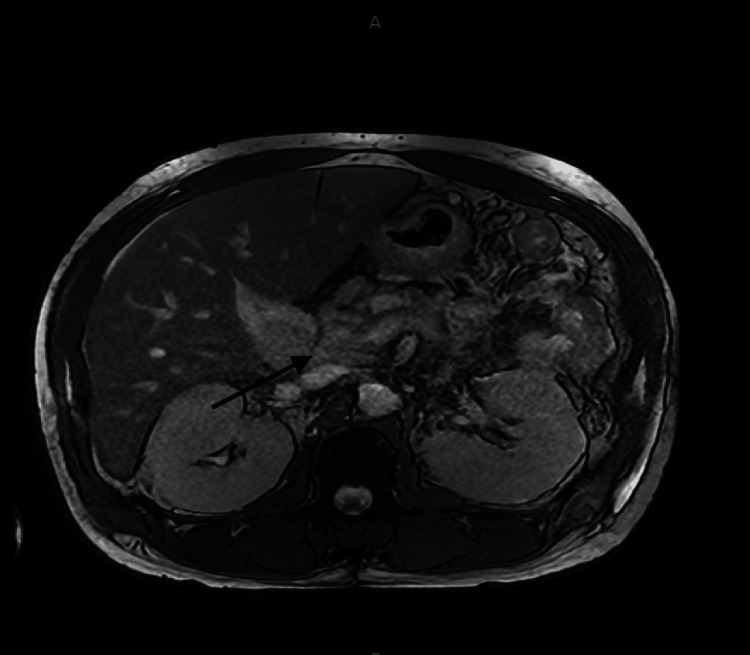
Axial cross-sectional abdominal imaging demonstrating peripancreatic inflammatory changes (black arrow) without portal vein thrombosis or hepatic infarction.

**Figure 3:**
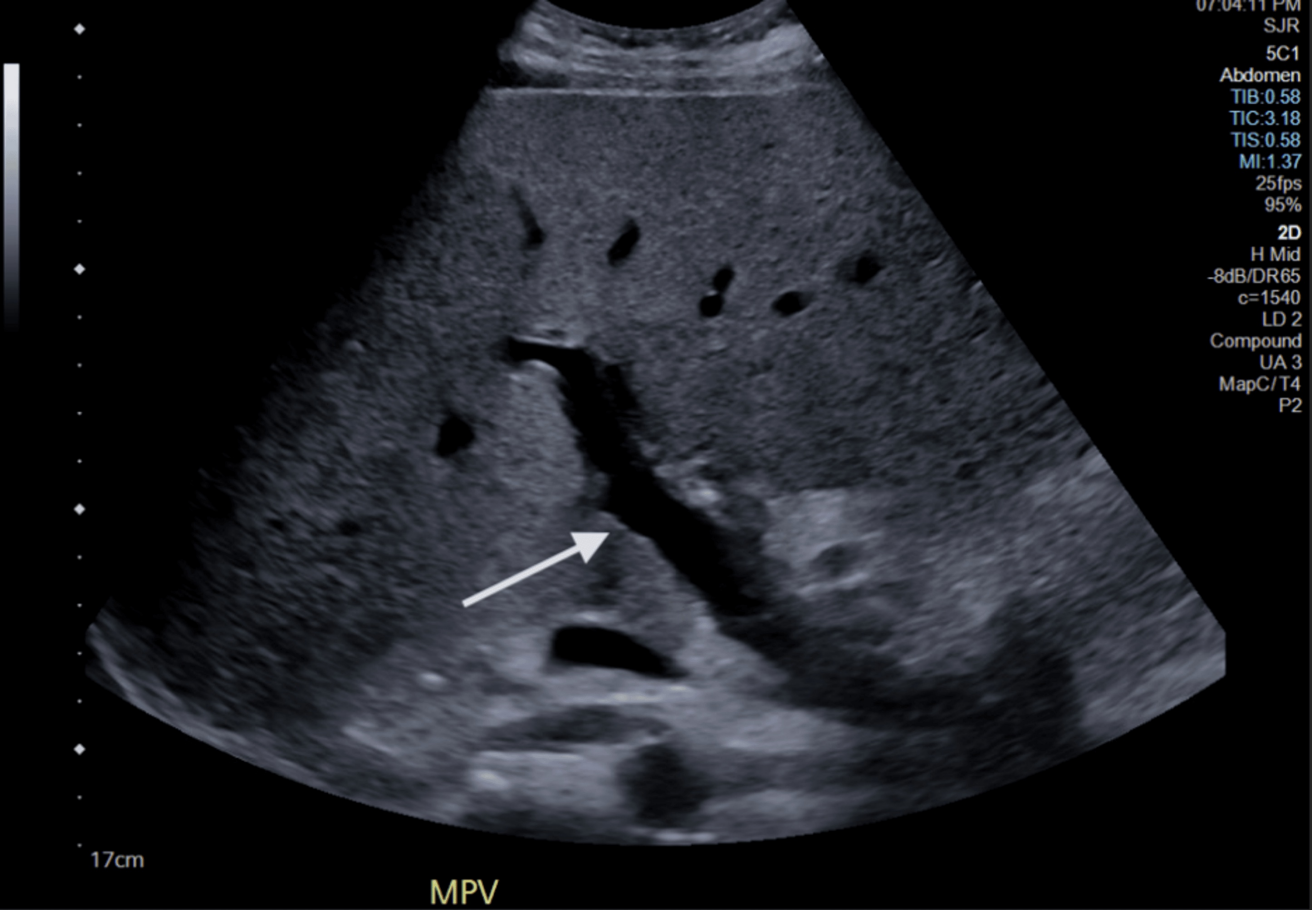
Abdominal ultrasound demonstrating patent main portal vein without thrombosis or flow obstruction (white arrow). Hepatic parenchyma demonstrated increased echogenicity consistent with steatosis. The absence of macrovascular occlusion supports microvascular ischemic hepatopathy as the mechanism of liver injury.

An extensive diagnostic evaluation was performed. Serial acetaminophen levels were within the normal range. Viral hepatitis serology, cytomegalovirus, and Epstein-Barr virus testing were negative, except for low-level Epstein-Barr virus transcripts. Autoimmune markers, including anti-smooth muscle antibody, were negative, and alpha-1 antitrypsin levels were normal. Abdominal imaging, including magnetic resonance imaging (MRI) of the abdomen (Figures 1, 2) and Doppler ultrasonography of the liver (Figure 3), demonstrated hepatic steatosis without evidence of vascular occlusion, biliary obstruction, or splenic infarction. Ferritin was markedly elevated (>5,700 ng/mL), with transferrin saturation exceeding 45%; however, HFE gene testing was negative. Ceruloplasmin levels were normal. Soluble interleukin-2 receptor was elevated at 2,453 pg/mL. Immunoglobulin G subclass testing was unremarkable, and haptoglobin levels were elevated.

**Figure 4:**
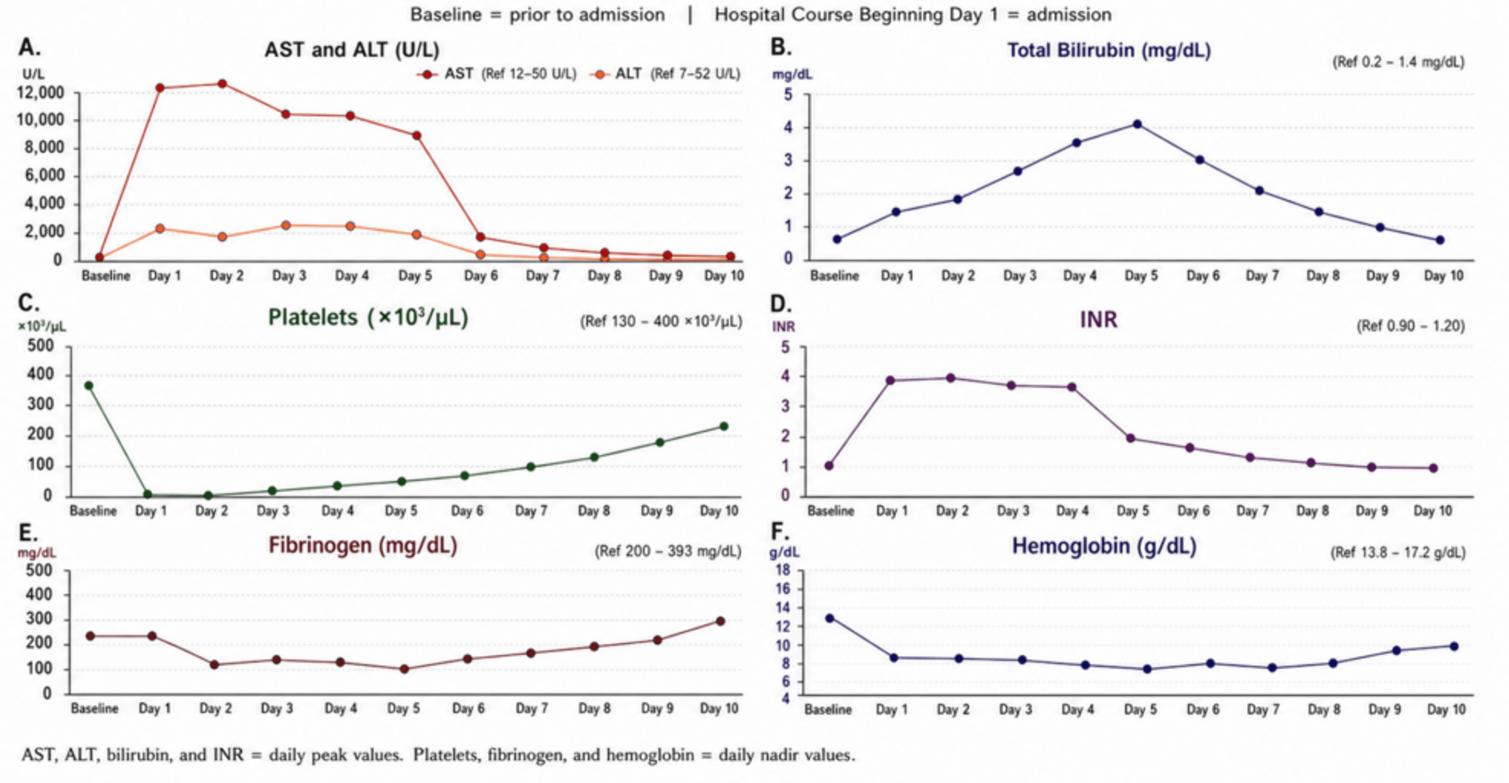
Laboratory trends observed during hospital course AST: aspartate aminotransferase, ALT: alanine aminotransferase, INR: international normalized ratio.

Figure 4 illustrates the patient's dynamic laboratory course from baseline through the first 10 days of hospitalization. Serum aminotransferase levels increased abruptly on admission, with AST rising from a baseline of 141 U/L to >10,000 U/L and remaining markedly elevated for approximately 48 hours before rapidly declining. In contrast, ALT increased from 44 U/L to a delayed peak of 2,216 U/L on day 2 and subsequently declined more gradually. Total bilirubin was initially within the normal range but increased progressively, peaking at 4.1 mg/dL on day 4 before steadily improving. Concurrently, severe thrombocytopenia developed, with the platelet count declining precipitously from a baseline of 361 × 10³/µL to a nadir of 4 × 10³/µL on admission, followed by progressive recovery to 221 × 10³/µL by day 10. Coagulopathy followed a similar course, with the INR increasing from a baseline of 1.02 to a peak of 3.81 on day 1 before gradually normalizing, while fibrinogen decreased from 220 mg/dL to a nadir of 108 mg/dL before recovering to 280 mg/dL by day 10. Hemoglobin declined from a baseline of 12.8 g/dL to a nadir of 7.6 g/dL before partially recovering to 9.8 g/dL by day 10. Collectively, these laboratory trends illustrate the evolution of severe ischemic hepatopathy accompanied by DIC, followed by gradual biochemical recovery with supportive management.

Table 1 summarizes the patient's key laboratory values at presentation and their corresponding peak abnormalities during hospitalization. 

**Table 1:** Key laboratory values at presentation and peak abnormality. AST: aspartate aminotransferase, ALT: alanine aminotransferase.

Laboratory test	Reference range	Baseline value	Peak/nadir value
AST (U/L)	12-50	69	>10,000
ALT (U/L)	7-52	40	2,216
Alkaline phosphatase (U/L)	32-126	107	322
Total bilirubin (mg/dL)	0.2-1.2	~1.0	~2.0
Platelets (×10⁹/L)	130-400	227	4
Ferritin (ng/mL)	30-400	-	>5,700
Soluble IL-2 receptor (pg/mL)	<1,033	-	2,453

Table 2 presents the diagnostic evaluation performed to determine the etiology of the patient's acute liver injury.

**Table 2:** Diagnostic evaluation for acute liver injury. CMV: cytomegalovirus, EBV: Epstein-Barr virus, PCR: polymerase chain reaction, TSAT: transferrin saturation, HLH: hemophagocytic lymphohistiocytosis.

Etiology considered	Diagnostic test(s)	Result	Interpretation
Viral hepatitis	HAV, HBV, HCV serologies	Negative	Excluded
Viral reactivation	CMV PCR, EBV PCR	CMV negative; low-level EBV	Unlikely primary cause
Autoimmune hepatitis	ASMA, IgG subclasses	Negative/normal	Excluded
Metabolic liver disease	Ceruloplasmin, Wilson testing	Normal	Excluded
Iron overload	Ferritin, TSAT, HFE testing	Ferritin high; HFE negative	Acute phase response
Vascular insult	Abdominal imaging	No infarction	Macrovascular ischemia excluded
Drug-induced liver injury	Acetaminophen levels	Acetaminophen levels normal	Excluded
HLH	HLH-2004 criteria	Criteria not met	Excluded

## Discussion

The magnitude and pattern of aminotransferase elevation in this case are most consistent with ischemic hepatopathy. AST or ALT levels exceeding 10,000 IU/L are uncommon and are typically associated with ischemic liver injury, acetaminophen toxicity, or severe viral hepatitis [1]. The relatively mild bilirubin elevation, absence of hepatic encephalopathy, and rapid biochemical recovery within one week strongly support ischemic hepatopathy [2]. Acute liver failure was strongly considered because of the markedly elevated INR in conjunction with the extreme transaminase elevation. However, the absence of hepatic encephalopathy and the rapid resolution of biochemical abnormalities argued against this diagnosis. Furthermore, the interpretation of the elevated INR was confounded by the presence of DIC.

Although the patient reported chronic acetaminophen use, the absence of a documented overdose, persistently normal serum acetaminophen levels, lack of progressive liver failure, and rapid normalization of liver enzymes argue against classic acetaminophen toxicity as the primary etiology. Imaging excluded large-vessel hepatic ischemia, suggesting a microvascular mechanism of injury.

Acute pancreatitis is known to activate systemic inflammatory and procoagulant pathways, predisposing patients to DIC and microvascular thrombotic complications [3,4]. The abrupt onset of severe thrombocytopenia, anemia, and coagulopathy supports a diagnosis of consumptive coagulopathy. We postulate that pancreatitis-associated DIC resulted in hepatic sinusoidal microthrombosis, leading to ischemic hepatopathy despite preserved systemic hemodynamics.

Severe acute pancreatitis (SAP) activates the coagulation cascade through several mechanisms. First, tissue factor (TF) expression on activated monocytes and the release of TF-bearing extracellular vesicles from necroptotic macrophages promote coagulation [5]. Second, proinflammatory cytokines impair endothelial anticoagulant pathways, particularly the protein C system, thereby facilitating thrombus propagation [6]. In addition, upregulation of plasminogen activator inhibitor-1 (PAI-1) suppresses fibrinolysis, preventing fibrin degradation and further exacerbating microvascular thrombosis [6]. The resulting uncontrolled thrombin generation leads to disseminated microvascular fibrin deposition, consumption of platelets and coagulation factors, and progression to overt DIC.

DIC-induced microvascular thrombosis within the hepatic sinusoids reduces oxygen delivery to hepatocytes, resulting in ischemic hepatopathy, also known as "shock liver" [7]. This condition typically manifests as a dramatic elevation in serum aminotransferase levels (more than 15-20 times the upper limit of normal), with bilirubin levels that are initially normal or only mildly elevated but may subsequently increase, often in parallel with worsening INR [8].

The liver is particularly vulnerable because it receives the first pass of pancreatic inflammatory mediators through the portal circulation, leading to Kupffer cell activation and amplification of the inflammatory response [9]. Furthermore, pancreatitis-associated ascitic fluid can directly induce hepatocyte apoptosis through activation of the p38-MAPK and caspase-3 pathways, independent of hemodynamic compromise. Concurrent hemodynamic instability in SAP may further exacerbate hepatic ischemia [10].

HLH was considered because of the markedly elevated ferritin and soluble interleukin-2 receptor levels. However, HLH was considered unlikely because the patient did not meet the HLH-2004 diagnostic criteria, lacking persistent fever, splenomegaly, cytopenias affecting multiple cell lineages, or clinical evidence of hemophagocytosis [11]. Furthermore, the rapid clinical and biochemical improvement occurred without immunosuppressive therapy, which would be atypical for HLH [12]. Hyperferritinemia and elevated soluble interleukin-2 receptor levels are recognized features of severe inflammatory states, including acute pancreatitis and DIC, and are not specific for HLH in isolation [13].

N-acetylcysteine was administered empirically. Beyond its established role in acetaminophen toxicity, N-acetylcysteine has demonstrated benefit in selected patients with non-acetaminophen acute liver injury by improving hepatic perfusion and reducing oxidative stress. Although it has not been specifically studied in ischemic hepatopathy, it is frequently administered in severe ischemic liver injury because of its potential to improve systemic and hepatic perfusion and may improve transplant-free survival [14]. Because the microvascular thrombosis occurred in the setting of DIC, treatment was directed toward the underlying cause, namely, acute pancreatitis. Routine anticoagulation is not recommended for pancreatitis-associated DIC and is contraindicated in patients with severe thrombocytopenia [15].

Although ischemic hepatopathy is typically reversible following correction of the underlying hemodynamic or microvascular insult, early prognostic assessment remains essential to identify patients who may progress to acute liver failure. The King's College Criteria are the most widely used prognostic tool for acute liver failure and may help guide timely referral to a liver transplant center. In non-acetaminophen acute liver failure, a poor prognosis is predicted by an INR > 6.5 or the presence of at least three of the following: age younger than 10 years or older than 40 years; jaundice for more than seven days before the onset of encephalopathy; INR > 3.5; serum bilirubin > 17.5 mg/dL (300 µmol/L); or an unfavorable etiology, such as Wilson disease or idiosyncratic drug-induced liver injury. Although these criteria were not developed specifically for ischemic hepatopathy, they remain useful for risk stratification in patients who develop acute liver failure. Because most cases of ischemic hepatopathy improve with treatment of the precipitating illness, liver transplantation is rarely required. However, patients with worsening hepatic synthetic dysfunction, hepatic encephalopathy, or failure to improve despite optimal supportive care should undergo early evaluation at a liver transplant center [16,17]. Our patient underwent liver transplant evaluation during hospitalization. In retrospect, however, he did not meet the diagnostic criteria for acute liver failure because he never developed hepatic encephalopathy, and the elevated INR was likely attributable, at least in part, to concurrent DIC.

To our knowledge, ischemic hepatopathy secondary to pancreatitis-associated DIC in the absence of systemic shock has been only rarely reported, highlighting the novelty and educational value of this case.

## Conclusions

This case highlights ischemic hepatopathy as a rare but important complication of acute pancreatitis that can occur in the absence of systemic hypotension. The characteristic pattern of profound aminotransferase elevation followed by rapid biochemical recovery, together with evidence of disseminated intravascular coagulation and consumptive coagulopathy, supports hepatic microvascular thrombosis and ischemia as the most likely mechanism of liver injury. Careful exclusion of alternative causes of acute liver injury, including viral hepatitis, drug-induced liver injury, autoimmune hepatitis, and vascular disorders, is essential to establish the diagnosis and avoid unnecessary interventions.

This case also emphasizes the importance of early recognition of ischemic hepatopathy in patients with severe inflammatory states, as localized hepatic hypoperfusion and sinusoidal microvascular thrombosis may produce a clinical presentation that is indistinguishable from other causes of acute liver injury despite preserved systemic blood pressure. Management should focus on treating the underlying precipitating illness and correcting the associated hemodynamic and thrombotic abnormalities, as hepatic injury is often reversible with appropriate supportive care. Patients who develop acute liver failure should undergo early evaluation at a liver transplant center. Although ischemic hepatopathy rarely requires liver transplantation because hepatic function often recovers after correction of the underlying insult, persistent coagulopathy, hepatic encephalopathy, or failure of liver function to improve should prompt urgent transplant assessment. Prognostic tools such as the King's College Criteria may aid risk stratification but should be interpreted within the context of the patient's overall clinical course.
